# Enhancing crop yield by using Rubisco activase to improve photosynthesis under elevated temperatures

**DOI:** 10.1007/s44154-021-00002-5

**Published:** 2021-08-18

**Authors:** Inosha Wijewardene, Guoxin Shen, Hong Zhang

**Affiliations:** 1 Department of Biological Sciences, Texas Tech University, Lubbock, TX 79409 USA; 2 Zhejiang Academy of Agricultural Sciences, Hangzhou, Zhejiang Province China

**Keywords:** Climate change, Drought, Heat stress, Temperature increase, Rubisco activase

## Abstract

With the rapid growth of world population, it is essential to increase agricultural productivity to feed the growing population. Over the past decades, many methods have been used to increase crop yields. Despite the success in boosting the crop yield through these methods, global food production still needs to be increased to be on par with the increasing population and its dynamic consumption patterns. Additionally, given the prevailing environmental conditions pertaining to the global temperature increase, heat stress will likely be a critical factor that negatively affects plant biomass and crop yield. One of the key elements hindering photosynthesis and plant productivity under heat stress is the thermo-sensitivity of the Rubisco activase (RCA), a molecular chaperone that converts Rubisco back to active form after it becomes inactive. It would be an attractive and practical strategy to maintain photosynthetic activity under elevated temperatures by enhancing the thermo-stability of RCA. In this context, this review discusses the need to improve the thermo-tolerance of RCA under current climatic conditions and to further study RCA structure and regulation, and its limitations at elevated temperatures. This review summarizes successful results and provides a perspective on RCA research and its implication in improving crop yield under elevated temperature conditions in the future.

## The issue at hand: the rising atmospheric temperatures

With the projected population to exceed 9 billion by 2050 and the global mean temperature rising by 1.5 °C, worldwide agricultural productivity needs to be increased by at least 60% or more amidst the changing climate, declining cropland and limited freshwater to sustain food production (Dubey et al. 2020). If the current trend of escalating temperature continues and goes up by 1.5 °C or more, the food productivity in the world, especially in the sub-Saharan Africa, Latin America, and parts of Asia, is expected to decrease, which would lead to serious consequences on food availability to people in those regions (Ipcc IPOCC and Bongaarts 2019). Therefore, despite that certain cooler regions of the world might benefit from this temperature increase, as a whole, the global agricultural scenario would still look negative in terms of productivity (Fahad et al. 2017).

Abiotic stresses such as drought, extreme temperatures (e.g. heat, chilling, and freezing), salinity, and floods account for more than 50% of the annual crop loss globally, which could increase to an even greater extent with the severity and frequency of adverse climatic changes (Minhas et al. 2017). Heat stress in particular leads to several consequences, for instance the soil temperature increasing due to high air temperature, subsequently causing a depletion in soil moisture, thereby exacerbating the severity of the heat stress (Akter and Islam 2017). Ultimately, critical activities of plants including seed germination, growth and development, pollen production, and photosynthesis are all unfavorably affected, resulting in poor crop yield (Rollins et al. 2013; Nadeem et al. 2018). For example, a recent study that evaluated 30 wheat cultivation sites across the world from 1981 to 2010 showed that there was a 1–28% and 6–55% yield reduction when there were temperature increases of 2 and 4 °C, respectively, indicating that for every 1 °C increase in atmospheric temperature, roughly 6% of yield reduction would be experienced in global wheat production (Akter and Islam 2017). Thus, it is imperative to investigate and explore strategies to improve agriculture production under heat stress conditions and select and breed thermo-tolerant cultivars as well as use molecular and genetic methods to create thermo-tolerant varieties that will be able to thrive and provide substantially higher yields under sub-optimal environmental conditions.

## Scope of genetic engineering in improving plant heat stress tolerance

Exploring the possibilities of improving plant biomass and productivity under elevated temperatures is vital to keep up with the demand of the growing population. In addition to unfavorable weather conditions such as heat stress, food production will be under the constraints of less water, less land, less labor, and less chemicals such as fertilizers and pesticides. The genetic engineering approach appears to be an effective alternative strategy to improve crop yield in the future alongside traditional breeding methods (Khush 2005; Nalluri and Karri 2020). Incorporation of the genetic engineering techniques could aid in overcoming several limitations of conventional breeding practices, and these limitations include the long time needed to introduce a new variety, the labor intensive screening of hundreds or thousands of varieties in replicated plot trials, and the introduction of undesirable traits along with the desirable characteristics (Ulukan 2009). Additionally, with smart farming or what is considered as the fourth revolution in agriculture, which utilizes information and communication technology to evaluate various field parameters such as biomass development calculation, soil and crop fertilizer status, irrigation requirement and potential soil leaching issues, sustainable agricultural practices will be a reality with the minimum use of resources instead of overexploitation (Walter et al. 2017). Therefore, adopting molecular approaches such as genetic engineering and gene editing to introduce thermo-tolerant crop varieties is an effective solution towards increasing crop yield through sustainable agricultural practices that would be worth of further exploration.

Among the numerous genetic engineering efforts tried over the years, overexpression of genes to enhance plant performance under moderate to high heat stress conditions has shown to be a successful approach (Singh et al. 2019). A variety of candidate genes have been introduced into different plants where varying degrees of increased heat tolerance have been achieved in the transgenic lines. The functions of the genes that were introduced into transgenic plants span across a diverse collection of activities such as transcription factors (Qi et al. 2018; El-Esawi et al. 2019), quantitative trait loci related to heat stress (Acuña-Galindo et al. 2015; Wen et al. 2019), heat shock proteins (HSPs)/chaperone proteins (Panzade et al. 2020; Wang et al. 2020), heat shock factors (HSFs) (Wang et al. 2017), osmolytes (Cvikrová et al. 2012), regulatory proteins participating in oxidative stress signaling (Lin et al. 2015; Ali et al. 2020; Ghosh et al. 2020), hormonal signaling (Bi et al. 2020), and abiotic stress response pathways (Zang et al. 2017; Lamaoui et al. 2018; Nadeem et al. 2018). Among these approaches, studies related to the photosynthetic machinery would seem like a winning strategy since the efficiency of photosynthesis ultimately determines plant biomass and yield, which makes sense in an economical scale and it directly translates into increased productivity (Antonovsky et al. 2017). At present, relatively limited work has been carried out in manipulating the components in the primary carbon assimilation pathway, i.e. the Calvin Benson Cycle enzymes, especially modifications to the key enzyme, ribulose-1,5-bisphosphate carboxylase-oxygenase (Rubisco) are scarce, due to the structural complexity of this enzyme complex, drawbacks of labor-intensive and time-consuming factors involved, the possibility of impairing plant growth and development, and deleterious consequences of the molecular manipulation (Wilson et al. 2019).

Although numerous studies have attempted to improve photosynthesis through methods such as overexpressing Rubisco subunits, and altering Rubisco properties or its catalytic features, enhanced photosynthetic rates could not be achieved (Suzuki et al. 2009; Wostrikoff et al. 2012). However, in a recent study involving maize overexpressing Rubisco small subunit (UBI-SS), both Rubisco large and small subunits (UBI-LSSS), the Rubisco assembly chaperone RUBISCO ASSEMBLY FACTOR 1 (RAF1) (UBI-RAF1), and Rubisco large and small subunits plus RAF1 (UBI-LSSS-RAF1), the results demonstrated that Rubisco content was significantly increased in UBI-LSSS-RAF1 plants compared to the other genotypes tested (Salesse-Smith et al. 2018). Moreover, the UBI-LSSS-RAF1 plants showed significantly higher biomass and longer height as well as approximately a 15% increased CO_2_ assimilation rate compared to that of wild-type plant, indicating the important role played by Rubisco chaperone proteins in improving photosynthesis. Also, the work conducted by Yoon et al. (2020) involving Rubisco overproducing (*RBCS*-sense) and Rubisco antisense (*RBCS*-antisense) rice plants, which had 130% and 35% of wild-type Rubisco levels, respectively, showed that dry weight, yield and nitrogen use efficiency (NUE) of the *RBCS*-sense plants were significantly increased upon sufficient nitrogen-fertilizer application, where the above parameters were higher at 15.0 Nm^− 2^ fertilizer application relative to 10.0 Nm^− 2^ fertilizer plot in the experimental paddy field (Yoon et al. 2020). In contrast, regardless of the N application, the yield of *RBCS*-antisense plants was significantly lower than wild-type rice plants. Additionally, the *RBCS*-sense plants displayed a greater biomass than wild-type plants at harvesting stage due to better NUE and higher N absorption, ultimately relating to improved yield, which was not shown by the *RBCS*-antisense plants, indicating that with sufficient nitrogen supply, the overexpression of Rubisco leads to enhanced photosynthesis per unit leaf area and improved yield. Nonetheless, considering the fact that the yield could not be improved under low N supply despite overexpression of Rubisco subunits only, it is implied that increasing yield and biomass of crops requires several contributors working together in order to bring a significant increase in productivity. Overuse of N in agriculture leads to various undesirable environmental consequences including eutrophication, while overproduction of Rubisco could cost plant to allocate more N for Rubisco, limiting or depriving other components in plant for access to nitrogen, which could eventually limit photosynthesis (Parry et al. 2003; Parry et al. 2007). Thus, it must be with great care and precision that plant productivity should be improved through interfering with the photosynthetic process, where exceptional understanding on photosynthesis is warranted to bring about a meaningful benefit in improving the plant yield via genetic engineering methods.

## Photosynthesis: a critical process affected by heat stress

Photosynthesis is a process that plays a critical role in plant growth and development, which directly translates into plant biomass and/or yield (Fan et al. 2018), while being one of the most heat sensitive cellular activities taking place inside chloroplast (Allakhverdiev et al. 2008). Heat stress could be described as an increase in temperature above a given limit that remains for a period of time, resulting in the irreversible damages in photosynthetic machinery, cellular structures and plant metabolism (Wahid et al. 2007). A heat shock or heat stress is created when the ambient temperature transiently rises by 10–15 °C, which could greatly affect the proper functioning of a plant. Depending on its duration, intensity, and rate of temperature increase, moderate to high heat stresses cause catastrophic events, directly affecting the photosynthetic process such as increased thylakoid membrane fluidity, aggregation, denaturation or degradation of proteins, and inactivation of enzymes, thereby compromising the overall efficiency of photosynthesis (Sharkey 2005). Consequently, exploring the possibilities of successfully introducing genes related to photosynthesis into plant genome to increase their thermo-tolerance holds great potential in enhancing photosynthesis-led increase in crop yield.

## Rubisco and photosynthesis

**Fig. 1:**
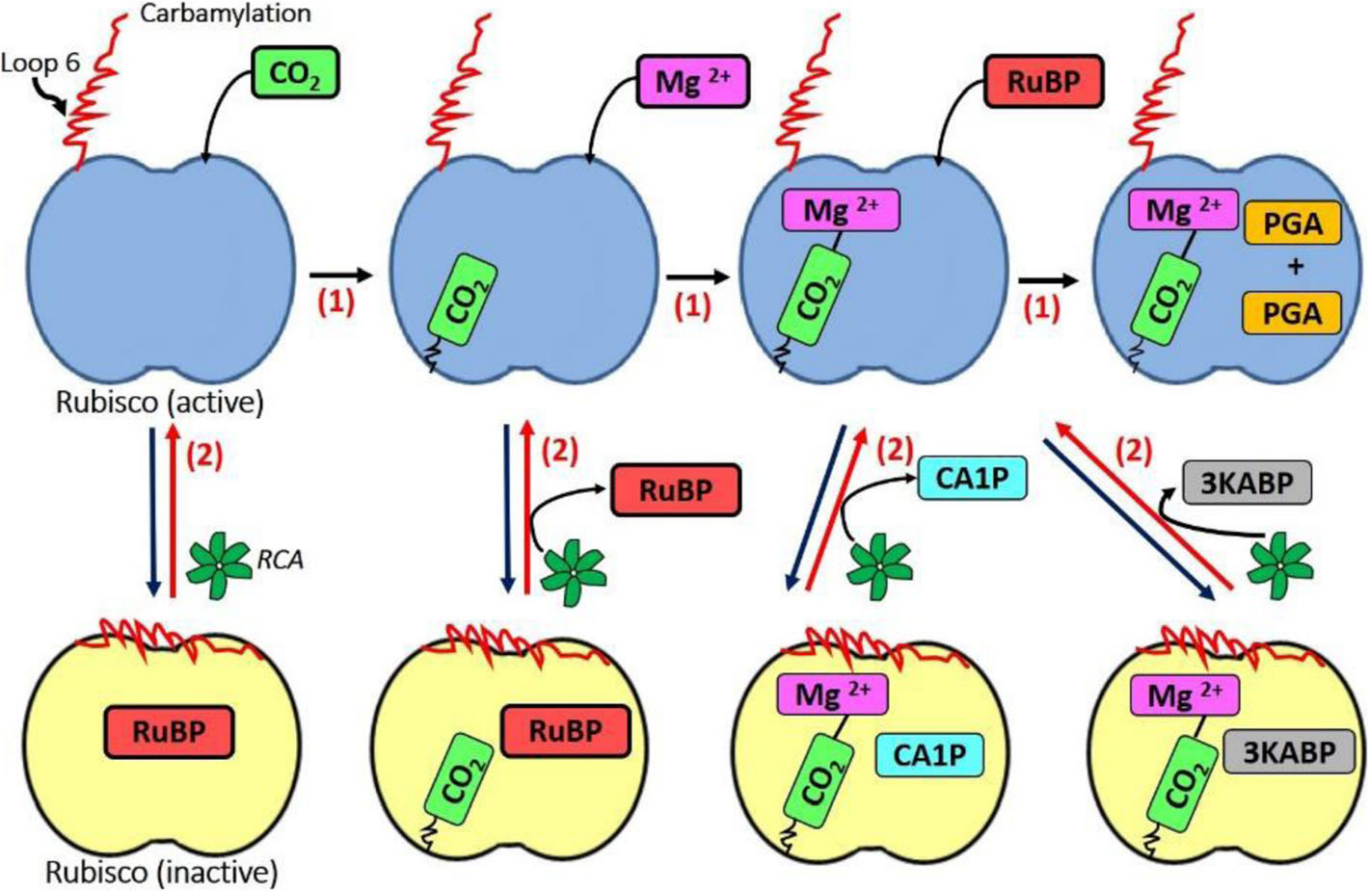
A model on the mechanism of activation of Rubisco by RCA. Under normal conditions (i.e. optimal temperature for RCA), the sequential entry of substrates, i.e. firstly CO_2_ (a process called carbamylation), then a divalent metal Mg^2+^, followed by ribulose 1,5-bisphosphate (RuBP), leading to the production of two molecules of 3-phosphoglycerate (PGA) by Rubisco (see upper sequential steps marked by 1). However, Rubisco can frequently become inactive by converting itself into a conformation called closed state (yellow colored Rubisco in the figure), especially if RuBP enters the active site first or other sugars such as 2-carboxyarabinitol 1- phosphate (CA1P) and 3-ketoarabinitol bisphosphate (3KABP) enter the active site, which could lead to the formation of the closed state. The function of RCA is to convert the closed state of Rubisco back to its active conformation, i.e. open state (reactions marked by 2), which allows the wrong molecules trapped in the active site to be released and the right substrates to enter the active site so that CO_2_ fixation could proceed normally

Under optimum conditions, CO_2_ binds to the active site of the most abundant protein on earth, Rubisco, in a process called carbamylation, followed by the binding of Mg^2+^, which is essential for the enzyme’s catalytic activity (Salvucci and Ogren 1996). Next, the substrate ribulose-1,5-bisphosphate (RuBP) binds to the carbamylated Rubisco (Fig. 1). However, various sugar phosphate molecules other than RuBP, could also bind to the active site of carbamylated or uncarbamylated Rubisco, leading to the formation of stable inactive forms of the enzyme-substrate complex (Fig. 1) (Portis Jr and Salvucci 2002). For example, certain inhibitory sugar phosphates, of which carboxyarabinitol 1-phosphate (CA1P) and 3-ketoarabinitol bisphosphate (3KABP) are the most common, could firmly bind to the carbamylated Rubisco, functioning as competitive inhibitors of RuBP, thereby preventing its interaction with the Rubisco active site (Fig. 1). Also, during the Rubisco reaction, molecules such as enediol intermediates get converted to XuBP (xylulose-1,5-bisphosphate) at the active site as a result of misprotonation, giving rise to catalytic misfires, a process which increases under heat stress (Salvucci and Crafts-Brandner 2004). Consequently, all these activities hinder the catalytic efficiency of Rubisco, causing a decrease in photosynthetic rate (Portis 2003). Interestingly, despite these obstacles Rubisco still exhibits 70–100% of activity, which was attributed to the activity of Rubisco activase (RCA) that allows Rubisco to efficiently function under physiological conditions (Portis Jr 1990). While Rubisco kinetics predominantly directs the photosynthetic efficiency at low temperatures, under high temperatures, both Rubisco kinetics as well as its active state determine the photosynthetic performance (Yamori et al. 2006).

Of vital importance is the fact that as the temperature rises beyond optimum, the rate of Rubisco inactivation increases than the rate of Rubisco activation driven by the RCA activity, leading to a net negative effect in Rubisco activation (Salvucci and Crafts-Brandner 2004). Stated simply, even though Rubisco, the principal enzyme in photosynthesis remains relatively stable at higher temperatures, the progressive loss of the ATPase activity of RCA due to its heat sensitivity, hampers the rate of photosynthesis as the temperature increases, causing a net effect of Rubisco inactivation under heat stress conditions (Law and Crafts-Brandner 1999; Crafts-Brandner and Salvucci 2000; Salvucci and Crafts-Brandner 2004b; Kim and Portis Jr 2005).

## The key player in Rubisco activation: Rubisco activase

Rubisco activase was initially discovered while conducting experiments to isolate photorespiratory mutants in *Arabidopsis thaliana*, through the identification of a nuclear gene mutant *rca* (regulation of carboxylase activation), where unlike the wild-type, the *rca* mutant could not activate Rubisco under *in vivo* conditions (Somerville et al. 1982). Upon further investigation, it was observed that the *rca* mutant was deficient of two polypeptides, roughly 47 and 43 kDa, relative to the wild-type in the two-dimensional polyacrylamide gel electrophoresis, in the soluble polypeptides from the chloroplast fraction of Arabidopsis plants (Portis Jr 1990). Subsequent experiments revealed that these polypeptides were essential in the Rubisco activation process (Somerville et al. 1982) and was later named as Rubisco activase (Salvucci et al. 1985). Corresponding to the different types of Rubisco enzymes such as green- and red-type, RCA has been identified in photosynthetic organisms from chemoautotrophic bacteria to dicots (Salvucci et al. 1987; Tsai et al. 2015; Loganathan et al. 2016). Rubisco activase is considered as a type of molecular chaperone (Demirevska-Kepova et al. 2005) that controls the activation and inactivation of another protein, since it binds to the substrate protein Rubisco, where through this controlled binding, RCA stabilizes and regulates the correct conformation of Rubisco, ensuring that sugar-phosphates are less tightly bound to Rubisco, enabling their release (Salvucci and Ogren 1996).

## RCA: structure, regulation, and its thermo-lability

RCA is a member of the AAA+ ATPases (ATPases associated with diverse cellular activities), displaying a core two-domain architecture across prokaryotic and eukaryotic RCA, with a flexible α/β-nucleotide binding subdomain at the N-terminus and an α-helical subdomain at the C-terminus (Shivhare and Mueller-Cajar 2017b; Flecken et al. 2020). RCAs of plants and green algae also contain a Rubisco recognition domain N-terminal to the AAA+ core while the ATPase activity is located near the extension at its C-terminus, which is critical for its activity as shown by the loss of function mutant analyses (Bhat et al. 2017b). RCA has a hexameric ring structure approximately 45 kDa in size, which requires a higher ATP/ADP ratio and free Mg^2+^ for accurate and efficient hexamer complex assembly (Kuriata et al. 2014). RCA commonly is made of two isoforms, arising either from a single gene via alternative splicing or separate genes (Werneke et al. 1989). The highly redox sensitive long isoform α (45–46 kDa) comprises a ~ 40 amino acid C-terminal extension that includes a pair of cysteine residues, while the shorter isoform β (41–43 kDa) shows no sensitivity to redox status (i.e. reduction/oxidation) (Shivhare et al. 2019). Several species including Arabidopsis, spinach, wheat, and rice have one *RCA* gene, whereas plants such as barley and cotton, sweet potato and tobacco contain two and three *RCA* genes, respectively (Salvucci et al. 2003; Jiang et al. 2014) (Table 1), although another research showed that cotton, too, contains three *RCA* genes (Law et al. 2001). While at least one gene encoding RCA is present in all plants, during evolution, gene duplication events resulted in several copies of *RCA* genes found in many plant species (Nagarajan and Gill 2018).

**Table 1:** Examples of RCA genes and isoforms characterized in several plant species

Plant	Number of genes	Number of isoforms	References
Arabidopsis (*Arabidopsis thaliana*)	One	Two or three: short isoform β (RCA1), long isoform α (RCA2)	(Zhang et al. 2001) (Kurek et al. 2007) (Kumar et al. 2009) (Deridder et al. 2012)
Spinach (*Spinacia oleracea*)	One	Two: short isoform (41 kDa) and long isoform (45 kDa)	(Werneke et al. 1989) (Shen et al. 1991) (Crafts-Brandner et al. 1997)
Rice (*Oryza sativa*)	One	Two: short isoform (RCA_S_, 43 kDa) and long isoform (RCA_L_, 47 kDa)	(To et al. 1999) (Zhang and Komatsu 2000) (Wang et al. 2010)
Wheat (*Triticum aestivum*)	Two	Three: short isoform TaRca2-β (42.2 kDa), long isoform TaRca2-α (46 kDa) and short isoform TaRca1-β (42.7 kDa)	(Carmo-Silva et al. 2015) (Kumar et al. 2016) (Bayramov 2017)
Maize (*Zea mays*)	Two	Two or three: two short isoforms β (41and 43 kDa) and a long isoform α (46.1 kDa)	(Martínez-Barajas et al. 1997) (Vargas-Suárez et al. 2004) (Yin et al. 2014) (Ayala-Ochoa et al. 2004)
Barley (*Hordeum vulgare*)	Two	Three: short isoform (42 kDa) and long isoform (46 kDa) from *RcaA*, third isoform (42 kDa) from *RcaB*	(Rundle and Zielinski 1991) (Rollins et al. 2013)
Cotton (*Gossypium hirsutum*)	Two or three	Three: short isoform (42–43 kDa), long isoform (46–47 kDa) and a third isoform (46 kDa)	(Law et al. 2001) (Salvucci et al. 2003) (Deridder and Salvucci 2007)
Sweet potato (*Ipomoea batatas*)	Two	Two: short isoform RCA (44 kDa) and long isoform RCAl (47 kDa)	(Xu et al. 2010) (Jiang et al. 2013) (Jiang et al. 2014)
Tobacco (*Nicotiana tabacum*)	Three	Three: short isoform β	(Qian and Rodermel 1993) (Carmo-Silva and Salvucci 2013) (Carmo-Silva et al. 2015)
Soybean (*Glycine max*)	Two	Two: short isoform β and long isoform α	(Yin et al. 2010)

Numerous *RCA* genes and isoforms have been studied in detail including spinach (Shen et al. 1991; Yamori et al. 2006), wheat (Law and Crafts-Brandner 2001), cotton (Salvucci et al. 2003), Arabidopsis (Salvucci et al. 2006), rice (Wang et al. 2010), maize (Yin et al. 2014), sweet potato (Jiang et al. 2013; Jiang et al. 2014), soybean (Chao et al. 2014), perennial ryegrass (Jurczyk et al. 2015a), and blue agave (Shivhare and Mueller-Cajar 2017a) (Table 1). In contrast, the regulation of *RCA* gene expression remains to be further explored as the research done so far has only barely revealed the complexity of its regulatory mechanism. Promoter sequences of *RCA* genes in several species have been investigated where a combination of *cis*- and *trans*-acting factors have been reported to control *RCA* gene expression in addition to external cues such as light (Watillon et al. 1993; Qu et al. 2011; Zhang et al. 2016).

Additionally, the roles RCA isoforms could be slightly different to each other, even though they all carry out Rubisco activation. For example, the rice RCA isoform α mediates photosynthesis acclimation to moderate heat stress while initial Rubisco activation under ambient temperature is carried out by the isoform β (Wang et al. 2010; Yin et al. 2014). In higher plants, *RCA* gene expression is leaf age and light dependent and organ specific, where it is largely expressed in the photosynthetic tissues (Watillon et al. 1993; Liu et al. 1996). Its expression is diurnal, where the beginning of the photoperiod shows the maximal transcript level and the minimal transcript level during the mid-day, followed by an upsurge in the dark period. RCA functions in removing Rubisco-bound inhibitory sugar phosphates at the expense of ATP hydrolysis (Carmo-Silva and Salvucci 2011; Carmo-Silva et al. 2015). Hence, under normal irradiance, the ATP/ADP ratio in the stroma is the chief regulator of the activase efficiency, however, if there is a co-expression of the two RCA isoforms, the chloroplast redox status is also involved in determining RCA activity (Ruuska et al. 2000; Zhang et al. 2002; Portis Jr et al. 2008; Henderson et al. 2011). Interestingly, in tobacco only the β isoform gene is expressed despite the critical role played by the α isoform in other plants in response to redox regulation and fluctuating light (Yin et al. 2014; Shivhare and Mueller-Cajar 2017b). However, the Arabidopsis transformant *rwt43* that contains only the Arabidopsis RCA isoform β, showed better sensitivity to ADP inhibition when tobacco RCA isoform β gene was expressed, indicating that the function and regulation of RCA is species dependent (Carmo-Silva and Salvucci 2013). Perdomo et al. (2019) reported that RCA-2β in wheat shows ADP insensitivity during Rubisco activation when compared to the other two wheat RCA isoforms that were inhibited by increasing amounts of ADP. Changing two amino acid residues in RCA-2β to resemble RCA-1β, caused the transformed RCA-2β to become ADP sensitive, suggesting the distinct properties of RCA isoforms, which directs them to respond to different physiological conditions. Moreover, work involving tobacco plants (*Nicotiana tabacum* L. cv Wisconsin 38) containing antisense DNA construct for *RCA* gene showed that reducing the total Rubisco activase content to less than 15% of the wild-type level led to a significant reduction in the Rubisco activation level during steady-state photosynthesis (Hammond et al. 1998). Additionally, the activase content became a limiting factor for Rubisco activation upon a rapid PFD increase. Thus, it could be possible that plants growing under continuous light and those growing under fluctuating irradiance may allocate relatively different amounts of proteins to Rubisco activase to maintain Rubisco activation. Recent studies report that in addition to redox regulation, post-translational modifications such as acetylation (Hartl et al. 2017) and phosphorylation (Kim et al. 2019) are also involved in controlling RCA activity. Several basic leucine zipper (bZIP) transcription factors were identified in soybean where the regulation of *RCA* gene expression appears to be trans-factor regulated (Zhang et al. 2016), opening doors to new areas of research to identify regulatory proteins in *RCA* gene expression in soybean as well as in other crops.

Thus, the roles of RCA in plant productivity are manifold: maintaining Rubisco in its active state to safeguard continuous photosynthetic efficiency and conferring acclimation to moderate heat stress during grain filling stage (Wang et al. 2010; Shan et al. 2011; Yin et al. 2014; Scafaro et al. 2016). However, the high thermo-lability of RCA makes it an unattractive player in photosynthesis at beyond-optimal temperatures. Several studies pointed out that weakened RCA activity or its interaction with Rubisco at elevated temperatures (> 30 °C) is one of the critical factors affecting photosynthetic rates under the present environmental conditions (Carmo-Silva et al. 2015; Busch and Sage 2017). Therefore, it would make sense to look into methods that would keep RCA active without itself being denatured and forming insoluble aggregates beyond the species-specific temperatures (Mueller-Cajar and Whitney 2008; Barta et al. 2010).

## Overexpression of *RCA*, a strategy with mixed results

Extensive research has been carried out pertaining to rice and improving its productivity under environmental stress conditions due to the fact that rice is consumed as a staple food by half of the world population (Shivhare and Mueller-Cajar 2017a; Liu et al. 2020). Unlike in cotton or wheat, the rice RCA isoforms are encoded by a single gene through post-transcriptional splicing of pre-mRNA, producing a 45 kDa isoform α and a 41 kDa isoform β, where the isoform α contains additional 33 amino acid residues at its C-terminus and five amino acid substitution immediately before the 33 amino acid residues (Scafaro et al. 2016). The relationship between rice RCA/Rubisco and photosynthetic activity has been intensively studied since 1990s till today, and it was reported that while RCA is closely linked with the rate of photosynthesis in leaves throughout their development, the *in vivo* Rubisco activity and RCA content had an inverse relationship especially at young leaf stages (Fukayama et al. 1996; Fukayama et al. 2012; Fukayama et al. 2018). In their initial experiments carried out on the 10th leaf arising from the main stem of the rice variety *Oryza sativa* L. cv. Nipponbare under saturating CO_2_ and light conditions, Fukayama et al. (1996) showed that, although the photosynthetic rate had a linear relationship with the RCA content, the linear relationship between Rubisco content and the rate of photosynthesis plateaued with Rubisco content beyond 3 g^− 2^ (Fukayama et al. 1996). Also, there was an exponential increase between Rubisco activity and the ratio of RCA/Rubisco. Further, while the maximum amount of Rubisco content in the leaf was seen by the third day of leaf emergence, the RCA content reached its highest amount by the 17th day, showing a greater accumulation of Rubisco in early leaf development compared to RCA. Yet, it was the RCA and photosynthetic rate, which had a linear relationship. Therefore, these findings led the authors to conclude that at the early leaf developmental stages, the rate of photosynthesis is not affected by Rubisco content higher than 3 g^− 2^, but positively responds to RCA, and the Rubisco activity is restricted by the amount of RCA.

Jin et al. (2006) introduced an antisense *RCA* into rice in which the RCA content was reduced to 30% of the wild-type level, and then investigated the localization of RCA as well as the alterations observed in Rubisco and RCA amount due to reduced RCA content. Key results from this work revealed that in rice, RCA was predominantly localized in the chloroplast stroma (~ 75%) with some in the thylakoid membranes (~ 25%), suggesting the species dependence of RCA localization as previously documented in *Amaranthus tricolor* (Hong et al. 2005) and spinach (Rokka et al. 2001). Compared to wild-type plants, there was a substantial increase in Rubisco amount in *RCA* antisense plants with more than 96% of Rubisco located in the chloroplast stroma, indicating that the increase in Rubisco content could offset the RCA deficit. Despite the total Rubisco activity being lower in wild-type plants than in *RCA* antisense plants, the net photosynthetic rate was significantly reduced in the *RCA* antisense plants. It was speculated that even though the amount of Rubisco increases when RCA is reduced, this could be to compensate for the loss of RCA, to have more Rubisco so the inactivation due to inhibitory sugar phosphate binding could be reduced although not completely outrun as shown by the decrease in the net photosynthetic rate.

In their more recent work using the *Oryza sativa* L. cv. Nipponbare overexpressing either RCA small isoforms of barley (*Hordeum vulgare* L. cv. Kashima) or maize (*Zea mays* L. cv. Golden Cross Bantam), Fukayama et al. (2018) reported that when compared to the wild-type plants, transgenic lines overexpressing the barley *RCA* small isoform had a higher activation of Rubisco. Further, irrespective of the nitrogen supply, transgenic plants showed the lowest rate of photosynthesis, had significantly reduced amounts of Rubisco in mature leaves (9th leaf) as well as fully expanded leaves, showed no significant down-regulation of the Rubisco gene expression, and had no significant negative effects on plant biomass, growth rate or yield (Fukayama et al. 2012). Successive work in this area involving transgenic rice overexpressing maize *RCA* (Ox-mRCA) and knockdown *rca* (KD-Rca) plants using antisense *RCA* confirmed their previous findings of negative relationship existing between RCA and Rubisco. Transgenic lines overexpressing maize RCA (Ox-mRCA) had higher levels of RCA and thus, lower levels of Rubisco compared to transgenic lines overexpressing barley RCA. There was a significant increase in the Rubisco content in the *rca* knockdown lines and the opposite in the *RCA*-overexpression lines, as was indicated in previous work involving antisense *RCA* (He et al. 1997; Jin et al. 2006). Also, this outcome was specific to Rubisco since there was no negative effect on enzymes in Calvin- Benson Cycle such as fructose1,6-bisphosphate aldolase, sedoheptulose-1,7-bisphosphatase and phosphoribulokinase due to overexpression or antisense *RCA* (Fukayama et al. 2018). Despite observing an inverse relationship between RCA amount and Rubisco/total leaf protein in this study, the qRT-PCR results displayed significantly higher transcript levels of Rubisco small subunit-encoding genes (i.e. *OsRbcS2*–*OsRbcS5*) and large subunit gene (*OsRbcL*), in both *RCA* overexpression as well as KD-Rca plants when compared to wild-type plants. This suggested a possible post-transcriptional regulation or degradative mechanism of Rubisco being responsible for the discrepancies observed at protein level between RCA and Rubisco. Additionally, during the polysome loading profile analysis where the distribution and sizes of the OsRbcS2, OsRbcL and Actin polysomes were analyzed, the results showed that OsRbcL translational activity was relatively higher in *RCA*-overexpressing lines compared to wild-type plants, while the *OsRbcS2* transcript profile remained similar in all genotypes tested. Therefore, it was implied that the negative relationship observed between RCA and Rubisco protein levels was not due to alterations in translation of these Rubisco small and large subunits. Collectively, considering the overall results and the possible mechanisms of how RCA negatively affects Rubisco content, these results indicated that RCA may be negatively affecting plant Rubisco content possibly by interfering with the post-translational regulatory mechanisms of Rubisco biosynthesis, although this hypothesis requires further validation.

Several other research related to RCA and Rubisco content explains this negative relationship from a different standpoint, where it is suggested that rather than a trade-off, the change in Rubisco amount observed in either Rubisco small subunit or *RCA* overexpression or antisense plants could come down to a matter of nitrogen allocation (Suganami et al. 2018; Suganami et al. 2020). As a result of overexpression of Rubisco small subunit genes, while the Rubisco content in the rice leaves increased in the transgenic plants, several other enzymes in Calvin Benson Cycle and RCA content decreased, despite this observation being somewhat conflicting to studies conducted in tobacco (Sage et al. 2008). The different results in tobacco and rice could be attributed to the difference in species since rice contains higher amount of Rubisco and a lesser repository of nitrogen, thereby having a higher impact in nitrogen partitioning (Suzuki et al. 2009; Suganami et al. 2018). Rice transgenic lines overexpressing different amounts of RCA driven by either Rubisco small subunit gene or *RCA* gene promoters were grown under varying nitrogen concentrations (Suganami et al. 2020). It was seen that, till a 50% increase of RCA than wild-type, there was no reduction in the Rubisco content in the transgenic plants compared to wild-type regardless of the nitrogen levels, while the decrease in Rubisco that was observed in *RCA* high-overexpressing lines could be offset with increasing nitrogen concentrations (Suganami et al. 2020). Thus, it was suggested that contradictory results obtained over several studies on the impact of RCA on Rubisco content (Mate et al. 1993; Eckardt et al. 1997; Yamori and Von Caemmerer 2009; Fukayama et al. 2018) is mainly due to dissimilarities in the nitrogen availability, since when *RCA* antisense plants were grown in low nitrogen concentrations, the Rubisco content in rice plants increased significantly (Suganami et al. 2020). Overexpressing both Rubisco subunit genes and *RCA* genes at moderate levels might prove to be a practical approach in improving photosynthesis, leading to better yield. In fact, several recent studies have shown that overexpression of both Rubisco small subunit (*RBCS*) and Rubisco activase (*RCA*) genes contributes toward enhancing photosynthesis in plants. For instance, a study where wild-type rice (*Oryza sativa*) along with rice Rubisco small subunit 2 (*RBCS2*) overexpressing (RBCS-ox), rice RCA small isoform overexpressing (RCA-ox), and Rubisco/RCA co-overexpressing (RBCS-RCA-ox) plants were used, showed that the CO_2_ assimilation rates at moderately high temperatures (32–36 °C) were highest in RBCS-RCA-ox plants (Suganami et al. 2021). Although the Rubisco amounts were slightly less in RCA-ox plants compared to other genotypes tested, the Rubisco activation state was higher in these plants at elevated temperatures, while all other plants tested showed a decrease in activation state of Rubisco as the temperatures were increased. Moreover, work carried out by Qu et al. (2021) using three rice lines wild-type, Rubisco activase overexpressing (oxRCA), and Rubisco/Rubisco activase co-overexpressing (oxRCA-RBCS) plants demonstrated that rate of CO_2_ assimilation (A) at 40 °C was 15% and 20% higher in oxRCA and oxRCA-RBCS plants, respectively, when compared to wild-type rice plants. Additionally, while there was no significant difference in the dry biomass between wild-type and oxRCA plants at 40 °C, the oxRCA-RBCS plants had a 26% higher dry weight compared to wild-type plants. Taken together, these findings provide evidence that co-overexpressing both Rubisco and RCA might prove to be a promising approach to improve photosynthesis and yield under elevated temperatures.

## Improving thermo-tolerance of RCA: initial work

Several early studies reported positive results through improving the thermo-tolerance of RCA, leading to enhanced photosynthesis at elevated temperatures. In one of these studies the thermo-stable RCA variants of *Arabidopsis thaliana* were generated through gene shuffling by isolating, fragmenting, and reassembling the RCA short isoform (RCA1), where these variants as well as wild-type RCA1 were expressed in the Arabidopsis *RCA* deletion mutant plant (Δ*rca*) that lacks *RCA* DNA sequence from exon 5 to 7 (Kurek et al. 2007). This was followed by exposing the transgenic lines to a continuously elevated temperature of 26 °C or 30 °C for 4 h a day (Kurek et al. 2007). Compared to the wild-type RCA1, the RCA variants maintained a higher Rubisco activity at elevated temperatures, indicating that RCA variants responded better than the wild-type RCA1 at moderate heat stress conditions. The homozygous *Δrca* plants showed distinct physiological and phenotypic characteristics compared to the wild-type plants, such as low photosynthetic rates at ambient CO_2_ concentration, lower leaf area and stunted growth. More importantly, a significantly higher number of siliques and higher seed weight were observed in the Δ*rca/RCA1* variant transgenic lines when compared to the wild-type and *Δrca/RCA1* heterozygous lines, when these plants were continuously exposed to 26 °C or 30 °C for 4 h a day. This research indicates the essential role played by RCA in photosynthesis and how improving its thermo-stability can lead to better plant growth and higher seed yield under moderate heat stress conditions.

Shortly after using the gene shuffling method, another approach was attempted in achieving the goal of enhancing RCA thermo-stability, i.e., producing a stable chimeric RCA to improve heat tolerance. Previous work have shown that RCA belonging to plant species growing in different temperature conditions have distinct cut off temperature optima for their activities (Salvucci et al. 2001; Salvucci and Crafts-Brandner 2004b). However, incorporation of a thermo-stable RCA in a different host plant had previously resulted in incompatibility due to species specificity, leading to a failure in activating Rubisco in the transgenic plants (Li et al. 2005). Kumar et al. (2009) created a chimeric RCA (i.e. Tob-Arab) where the Rubisco recognition domain of tobacco RCA was replaced with the one from Arabidopsis RCA, which was expressed in the Arabidopsis *rca* mutant deficient of wild-type RCA (Kumar et al. 2009). *In vitro* studies showed that after 20 min at 37 °C, Tob-Arab retained 83% of its initial activity at 25 °C, compared to the wild-type Arabidopsis and tobacco that retained 33% and 67% of their initial activities, respectively (Kumar et al. 2009). Similar trend was observed at 40 °C for 20 min, where wild-type Arabidopsis RCA showed minimal activity, while tobacco and Tob-Arab preserved 40% and 50% of their initial activities, respectively. Further, after 6 weeks of growth in continuous temperature at 27 °C, transgenic plants expressing the chimeric RCA displayed greater biomass than wild-type plants, while the photosynthetic rate after a brief exposure to 38 °C, was 38–46% greater than that of wild-type plants. As a result of higher rates of photosynthesis and improved biomass, the transgenic lines produced about four times more total seed weight per plant than wild-type plants, as well as higher seed viability. These findings further strengthened the notion of improved plant productivity due to better thermo-tolerance of RCA.

In addition to these research pertaining to RCA and its enhanced thermo-tolerance, numerous studies in the past decades provided us with better insight into RCA through identification and characterization of its subunits and isoforms as well as molecular methods that could be adopted to improve the thermo-tolerance of RCA to increase plant productivity. As of now, the crystal structures of RCA from three different plant species, tobacco (Stotz et al. 2011), desert shrub creosote (Henderson et al. 2011), and Arabidopsis (Hasse et al. 2015), have been elucidated. However, a major drawback that hampers the study of RCA proteins is the difficulty in isolating this enzyme in its native form with the complete amino acid sequence due to its conformational flexibility and instability, leading to the formation of large aggregates during protein purification (Hasse et al. 2015). Thus, only partial amino acid sequences or the truncated forms of RCA were analyzed in the study of its crystal structure and in predicting the exact mechanism of Rubisco-RCA interaction. However, a completely functional RCA was recently engineered using the photosynthetic proteobacterium *Rhodobacter sphaeroides*, which allowed the study of the protein-protein interactions between Rubisco and RCA under normal conditions (Bhat et al. 2017a). This has provided a better insight into how the Rubisco catalytic core and its vicinity interacts with or binds to RCA in Rubisco activation, facilitating RCA to open the Rubisco active site. Similar findings have been reported where it is shown that RCA top surface acts as the Rubisco interacting face, with the RCA α4-β4 loop contributing towards the RCA-Rubisco interaction (Shivhare and Mueller-Cajar 2017a). This work has broadened the understanding of RCA function in Rubisco activation, which is invaluable in unraveling the detailed mechanism on the conformational changes taking place in Rubisco activation, while also helping to explore the structural and biochemical tweaks required to improve Rubisco carboxylation as well as RCA thermo-tolerance in crop plants.

## RCA and heat acclimation

When plants are exposed to prolonged and gradual increase of a particular stress such as heat, low temperature or water deficit, certain physiological and biochemical changes develop in plants to tolerate these stresses to a certain extent, so that plants could perform better under a new environmental condition, which is coarsely described as acclimation (Anjum 2015; Sándor et al. 2018). Therefore, many studies have explored RCA’s capacity in heat acclimation, due to its thermo-lability. When creeping bentgrass (*Agrostis stolonifera* L.), a type of cool-season perennial grass, was exposed to either gradual temperature increase from 20, 25, 30, 35 to 40 °C, or immediate increase from 20 to 40 °C, the heat acclimated plants displayed significantly better photosynthetic rates, higher Rubisco activity and activase activities (Liu and Huang 2008). Hence, unlike in a rapid increase in the environmental temperature where RCA tends to undergo thermal denaturation and form insoluble aggregates, when plants are exposed to a slow incremental temperature, it enables RCA to get acclimated to the increasing heat stress (Salvucci and Crafts-Brandner 2004a; Salvucci 2008; Deridder et al. 2012). RCA undergoes heat acclimation using many strategies including enzyme stabilization, producing heat-stable or heat-inducible isoforms, or increasing or decreasing the isoform expression levels depending on their sensitivity to heat stress, resulting in a RCA isoform ratio different to that of under normal conditions (Portis 2003; Kurek et al. 2007; Salvucci 2008). A study demonstrating the role of RCA in keeping Rubisco active was conducted when tobacco plants transformed with the *RCA* antisense construct and wild-type tobacco plants were exposed to heat stress, where the photosynthetic rates declined above 30 °C and 36 °C, respectively, while wild-type plants showed a significantly higher recovery rate in photosynthesis upon decreasing the temperature compared to the antisense plants (Sharkey et al. 2001).

Within this frame, many research have focused on the relationship of RCA isoforms and regulatory elements under heat stress, which underscores the importance of improving the thermo-tolerance of plants through acclimation and how RCA could contribute towards increased productivity under heat stress conditions. For instance, in many crops including wheat, cotton and maize, a heat inducible RCA isoform was detected along with the isoforms that are constitutively expressed (Deridder and Salvucci 2007). Earlier work on cotton reported a new RCA isoform of 46 kDa in addition to the constitutively expressed 47 and 43 kDa polypeptides, being synthesized upon exposure to 41/37 °C, which was degraded after removing the heat stress (Law et al. 2001). Such emergence of new RCA isoforms that are not constitutively expressed were found in several monocot crops including maize (Sanchez De Jimenez et al. 1995) and wheat (Law and Crafts-Brandner 2001). The third RCA isoform in cotton upon exposure to heat stress declined drastically although recovered during prolonged heat stress, and the longer transcripts of α isoforms *GhRCAα1* and *GhRCAα2* showed five different length variations at their 3′ untranslated regions (3′-UTRs), specifically lacking the putative instability sequences, indicating the possibility of maintaining the RCA transcript stability during extended heat stress, which could be attributed to a post-transcriptional mechanism contributing to photosynthesis acclimatization (Deridder and Salvucci 2007). Work on Arabidopsis RCA showed that among the three isoforms under heat stress conditions, the abundance of *AtRCAβ2* isoform tripled from 4% to 12% of the total *RCA* pool, leading to an overall reduction of the Arabidopsis *RCA* average 3′-UTR length since *AtRCAβ2* contains the shortest invariable 3′-UTR length among isoforms, supporting a previous idea that shorter 3′-UTR length confers better RCA thermo-stability (Deridder et al. 2012). Further, qPCR analysis of the isoforms showed that the *AtRCAβ2* transcript level remained stable throughout heat stress treatment, while the transcript levels of *AtRCAα* and *AtRCAβ* were relatively less stable, implying the importance of *AtRCAβ2* in maintaining steady-state *RCA* transcript levels under heat stress conditions.

Previous work on wheat RCA under heat stress condition indicated an increase in one of the constitutive RCA isoform of 42 kDa with the larger isoform of 46 kDa showing no increase, while a putative 41 kDa isoform transcript was induced upon exposure to heat stress (Law and Crafts-Brandner 2001). In contrast, more recent studies showed that, when compared to the 42 kDa isoform, a higher abundance of the 45–46 kDa isoform was observed when wheat plants were exposed to heat stress conditions at 36/30 °C (day/night) for 7 days, where a positive association between the higher transcript level of this wheat isoform and plant biomass and productivity was also reported (Ristic et al. 2009; Kumar et al. 2016). However, when barley was exposed to heat stress at 36 °C, there was a significant upregulation of the Rubisco activase B transcript while the Rubisco activase A transcript was downregulated (Rollins et al. 2013). Additionally, transcriptome analysis using Next Generation Sequencing was performed on thermo-tolerant and thermo-susceptible wheat varieties under control and heat stress conditions, where eight putative *RCA* transcripts were identified and a novel gene *TaRCA1* was cloned (Kumar et al. 2016). This study confirmed the findings associated with the previous work and transcriptome analyses involving wheat, where the thermo-tolerant varieties showed higher RCA expression, greater total antioxidant capacity, increased Rubisco activity and an increased RCA activity under heat stress conditions (Xue et al. 2015; Kumar et al. 2017; Kumar et al. 2019). The findings also pointed out that at different developmental stages, the expression patterns of *RCA* genes differed among the wheat varieties, influencing the accumulation of Rubisco, which mirrored the *RCA* transcript dynamics, showing the importance and potential of TaRCA1 in regulating Rubisco activity and how it could be used for the benefit of enhancing thermo-tolerance in crops. This also shows how the thermo-stability of RCA depends on the species and the climatic and environmental conditions in which it has evolved.

A study on red maple using heat sensitive “Northwood” and heat insensitive “Florida flame” showed similar results, where the CO_2_ assimilation rates dropped at temperatures above 33 °C in the heat sensitive “Northwood” with an increase observed in small RCA isoform, while it was beyond 40 °C for the heat insensitive “Florida flame” with both isoforms showing a modest increase, implying that though not solely responsible for the thermo-tolerance displayed, RCA could contribute substantially to the heat acclimation (Weston et al. 2007). It was found that within the same species, the amino acid sequences of RCA and Rubisco could vary significantly, giving rise to thermo-tolerant and thermo-susceptible varieties (Kumar et al. 2019). As shown in the transcriptome data, within the same crop wheat, there are thermo-tolerant and thermo-susceptible varieties that display varying degrees of RCA activity under heat stress conditions. Recently, a conserved sequence in RCA was identified in a thermo-tolerant wheat species that confers heat stability (Scafaro et al. 2019). The response of the three wheat RCA isoforms, TaRCA1-β, TaRCA2-α, and TaRCA2-β, to heat stress was first analyzed through expression profiling, followed by linking the association between regulation of these isoforms and RCA thermo-stability during heat stress treatment. It was observed that, similar to cotton and maize, expression of more thermally stable RCA isoforms is induced in response to heat stress in wheat, in particular the *TaRCA1*-*β* gene irrespective of the cultivar. Additionally, the amino acid sequence from the thermally susceptible TaRCA2-β was compared with TaRCA1-β, the rice RCA isoform OsRCA-β, and cold- and warm-adapted species’ RCA consensus sequences using sequence alignment and protein structural modeling. There were 11, 8 and 3 amino acid substitutions made in the wild-type TaRCA2-β amino acid sequence, based on the differences identified exclusively with TaRCA1-β, OsRCA-β, TaRCA2-β, and/or cold-adapted RCA. These three types of amino acid variants were named as TaRCA2-β-11AA, TaRCA2-β-8AA and TaRCA2-β-3AA. The subsequent experiments showed that all three TaRCA2-β variants had higher thermo-stability than did wild-type TaRCA2-β isoform, where TaRCA2-β-11AA displayed the greatest thermo-stability at temperatures of 41.5 °C to 42.4 °C, which was 7 °C more than wild-type TaRCA2-β. Further, the kinetic efficiency of the TaRCA2-β mutants did not appear to be affected due to the amino acid alterations, showing great potential of utilizing this technique to improve the RCA thermo-tolerance without compromising the enzyme efficiency. A recent study on wheat showed how a single point mutation created in RCA2β at position 159 to replace methionine with isoleucine, conferred enhanced thermo-tolerance of wheat RCA activity while also sustaining the Rubisco activation state (Degen et al. 2020). Likewise, biochemical studies on agave and rice RCA isoforms have revealed that due to differences in the amino acid sequences at the N-terminus of agave RCA, it is 10 °C more thermo-stable than rice RCA, while also maintaining a relatively higher ATPase and activase activity at low temperatures such as 15 °C (Shivhare and Mueller-Cajar 2017a). Thus, either overexpression of the thermo-stable RCA isoforms or editing the thermo-labile isoforms to improve their thermo-stability could prove to be practical approaches in achieving thermo-tolerance of plants.

However, a broader knowledge of RCA regulation related to its subunit composition and amount is necessary in order to effectively improve its thermo-tolerance and reap its benefits in agriculture. Regulation of RCA expression in plants does not always share the same mechanism, for example, transcriptional mechanisms leading to transcript abundance changes were observed in cotton, whereas translational mechanisms were apparent in wheat (Law and Crafts-Brandner 2001; Deridder and Salvucci 2007). In maize, the amount of RCA polypeptide and chaperon activity are regulated post-translationally with developmental and environmental cues playing a significant role (Vargas-Suárez et al. 2004). Moreover, some early research also showed contradicting results with different isoforms having a higher abundance under heat stress such as in wheat, which could be due to the discrepancies existing among many factors including experimental conditions, plant age or developmental stages (Law and Crafts-Brandner 2001; Ristic et al. 2009). In different crops, depending on the type of stress imposed, the abundance of specific RCA isoform varies (Perdomo et al. 2017), which further adds to the complexity of RCA expression and regulation, highlighting the need to understand RCA, Rubisco, and their interaction at elevated temperatures.

Rice RCA isoforms were shown to be unequally localized in different parts of the leaves, leading to distinct photosynthetic rates and participating differently in photosynthetic heat acclimation. The work by Wang et al. (2009) using *Oryza sativa* L. ‘Zhenong 952’ reported that under control conditions, the larger RCA isoform (RCA_L_)/smaller isoform (RCA_S_) ratio in fully expanded leaves was 1:5, which is likely due to transcript levels of the two isoforms, corroborating with their subsequent work on investigating the functions of the different rice RCA isoforms (Wang et al. 2010). Although there were differences in the isoform protein levels in leaves at varying developmental stages, the mRNA content corresponding to these isoforms showed no significant difference, pointing toward a post-translational regulation of RCA isoforms rather than transcriptional (Wang et al. 2009). Their initial work showed that the leaves with the highest photosynthetic rates at 35 °C had a higher RCA_L_/RCA_S_ ratio, while the oldest leaf (leaf 2) with the lowest photosynthetic rate had a lower RCA_L_/RCA_S_ ratio (Wang et al. 2009). They also demonstrated that upon exposure to a heat stress cycle of 40/30 °C, the alternative splicing of RCA pre-mRNA produced more RCA_L_, resulting in a significantly higher amount of RCA_L_ vs RCA_S_ during heat stress treatment. Once the heat stress was removed, the RCA_L_ and RCA_S_ levels returned to the levels detected under control conditions again (Wang et al. 2010). Additionally, the transgenic rice overexpressing *RCA*_*L*_ had better photosynthetic rates during heat stress treatment compared to wild-type and *RCA*_*s*-_overexpression lines. The latter showed a better photosynthetic rate and initial Rubisco activity under control conditions, as well as a higher amount of RCA_S_ isoform in rice leaves under controlled condition, implying the different roles played by the two RCA isoforms under different conditions, which could be applicable in generating thermo-tolerant crop varieties.

In addition to more thermo-stable isoforms, in recent years several rice varieties have been identified that displayed better heat tolerance when compared to its most common species *Oryza sativa*. Thus, when the commercial *O. sativa* (*Os*) and two wild rice species *Oryza meridionalis* Ng. (*Om*) and *Oryza australiensis* Domin (*Oa*) were exposed to heat stress conditions (i.e. 45/28 °C), *Oa* plants showed the greatest leaf elongation rates, the highest Rubisco activation state during heat stress treatment and no significant decrease in the net photosynthesis (*P*_*n*_), while *P*_*n*_ was reduced by roughly 40% and 27% under control conditions in *Os* and *Om*, respectively (Scafaro et al. 2016). Further, the purified extracts of RCA isoform β of *Oa* remained active at 42 °C, while the RCA from *Os* was inhibited at temperatures beyond 34 °C. Probing into the DNA and amino acid sequences of the RCA in *Os* and *Oa* revealed that, the differences observed between the two were primarily located toward the N- and C-termini, which could explain why the RCA from *Oa* appeared to be more thermo-stable. The most unstable flexible regions of the RCA are the C- and N-terminal domains that are critical in Rubisco recognition and binding (Li et al. 2005; Stotz et al. 2011; Parry et al. 2013), hence the differences existing in the RCA from *Oa* in this region compared to that of *Os* likely lead to better stability and reduced structural flexibility, thereby causing it to be more thermo-stable while also decreasing the species specific activity, although the latter was compensated by the higher ratio of RCA to Rubisco observed in *Oa* (Scafaro et al. 2016). Research on agave RCA structure also showed that when compared to the RCAs of tobacco and Arabidopsis, agave RCA contains an additional 11 amino acid residues in a loop that connects the alpha helix 4 and beta strand 4, which aids in Rubisco interaction. This leads to less structural flexibility, thereby conferring a higher thermo-stability to agave RCA (Shivhare and Mueller-Cajar 2017a). An extension to the rice RCA work was carried out where plant growth, photosynthesis and yield were evaluated in transgenic *O. sativa* lines overexpressing different levels of RCA isoforms of *Oa* (e.g. 9–19% of the total plant RCA content) under heat stress conditions (45/22 °C) (Scafaro et al. 2018). Results show that while plant height and biomass between the transgenic and wild-type plants showed no major difference, the number of seeds at maturity in the transgenic plants containing 19% of *Oa*’s RCA in the total RCA pool were around 2.5 fold higher than that of wild-type plants under heat stress conditions, indicating that the transgenic plants benefited from the improved Rubisco activation kinetics and non-steady-state photosynthesis under heat stress due to overexpression of a thermo-tolerant RCA.

## Overexpression of *RCA* for enhanced thermo-tolerance: recent progress

**Fig. 2:**
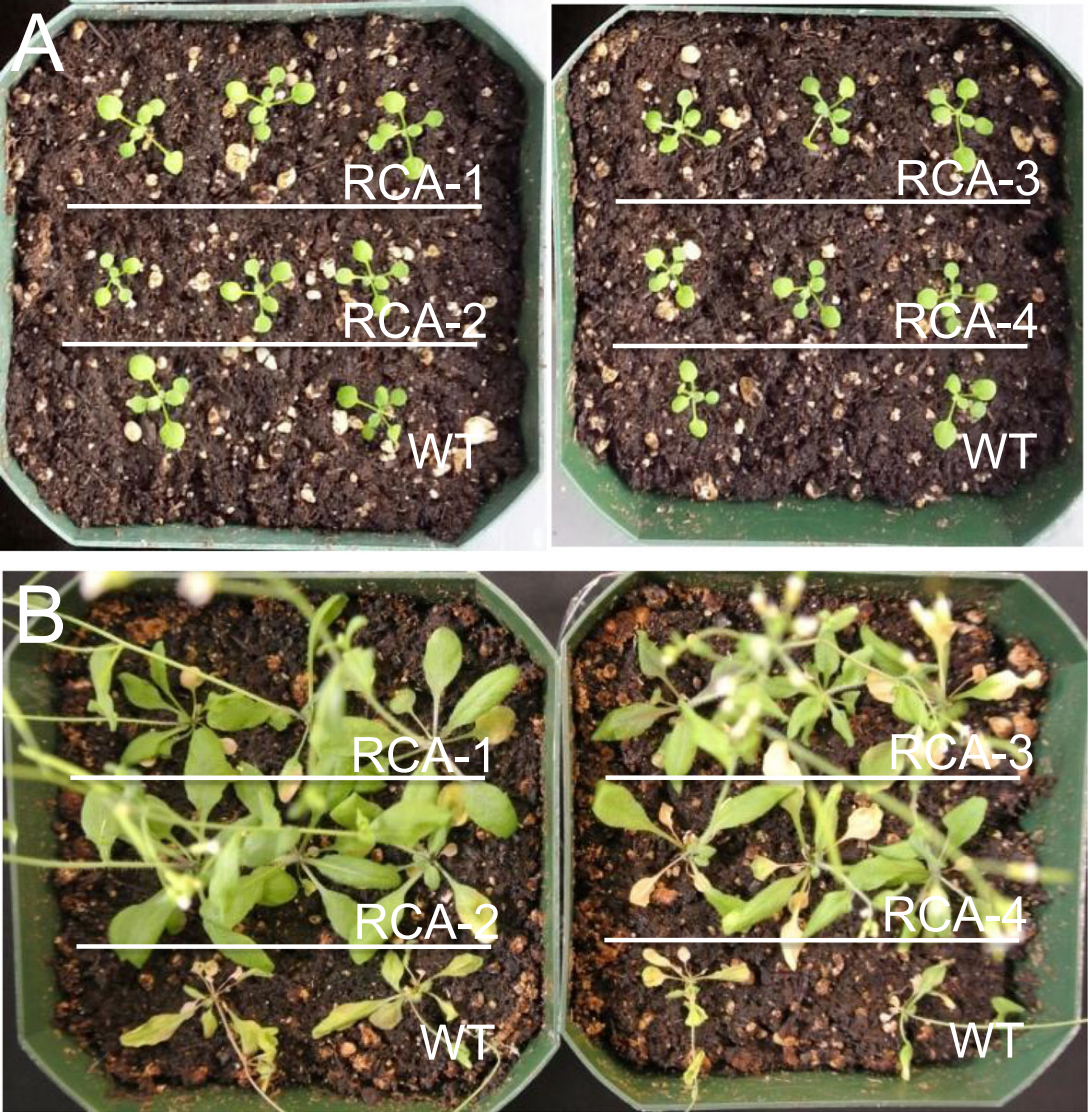
Phenotypes of *LtRCA*-overexpressing plants under heat stress conditions. **a** Arabidopsis plants before heat stress treatment. **b** Arabidopsis plants after heat stress treatment. Arabidopsis plants were grown under normal growth condition (22 °C continuously) for 3 weeks (photo A was taken at day 20), then moved to a heat chamber (42 °C for 5 h and 22 °C for 19 h each day) until the end of the experiment (photo B was taken at day 40 after the start of heat treatment). WT, wild-type plants; RCA-1 to RCA-4, four independent *LtRCA*-overexpressing plants

In recent years, research was focused on overexpressing different *RCA*s in a variety of plant species to ascertain their ability to enhance thermo-tolerance, thereby improving photosynthetic rates and plant productivity under elevated temperatures. Compared to the wild-type plants, *RCA*-overexpressing cucumber displayed significant improved thermo-tolerance at 40 °C, with higher mRNA abundance of Rubisco subunit genes, more Rubisco enzyme, and higher RCA activities, leading to better photosynthetic rates (Bi et al. 2016). To test if overexpressing a naturally more thermo-tolerant RCA in transgenic plants could improve photosynthesis, thereby leading to higher seed yield under heat stress conditions, we overexpressed the *RCA* isoform α gene, *LtRCA*, from a desert shrub commonly growing in Arizona and Mexico (i.e. *Larrea tridentate*) in Arabidopsis and analyzed how *LtRCA*-overexpressing Arabidopsis plants would perform under heat stress conditions. The rationale behind this experiment was based on the fact that *Larrea tridentate* has adapted to the hot desert in America’s Southwest, and it frequently experiences temperatures above 40–45 °C in the summer, therefore if the RCA from *Larrea tridentate* would interact with the Rubisco in Arabidopsis, then it might improve the performance of Arabidopsis’ Rubisco under heat stress conditions. Indeed, we found that *LtRCA*-overexpressing Arabidopsis plants performed much better than wild-type plants under the heat stress condition of 42 °C for 5 h and 22 °C for 19 h per day (Fig. 2b). It appears that *LtRCA*-overexpression only affects the heat tolerance, which was expected as RCA only affects Rubisco’s activity (i.e. CO_2_ fixation), not other cellular metabolic functions.

In nature, abiotic stresses rarely come alone, instead they often come in various combinations, especially for drought and heat stresses. Therefore it would make more sense if we make plants more drought and heat tolerant simultaneously, which could dramatically increase yield for crops grown in arid and semi-arid regions of the world. We then tested the idea of co-overexpression of *LtRCA* with another gene that confers increased drought- and salt-tolerance in order to obtain transgenic plants that are significantly more heat-, drought-, and salt-tolerant. For this purpose, we stacked the *LtRCA* gene with the *AVP1* gene from Arabidopsis and introduced these two genes into Arabidopsis. Previous work indicated that overexpression of *AVP1* in transgenic plants could improve both drought and salt tolerance (Gaxiola et al. 2001; Pasapula et al. 2011), consequently we expected that *AVP1/LtRCA* co-overexpressing plants would be more tolerant under heat, drought, salt, as well as under multiple stress conditions. Indeed, this was exactly what we observed. For example, when wild-type and *AVP1/LtRCA* co-overexpressing plants were exposed to heat stress conditions (37/22 °C for 5/19 h per day) for 2 months, *AVP1/LtRCA* co-overexpressing plants were indeed more tolerant to heat, drought, and salt stress separately as well as in all combinations (Wijewardene et al. 2020). More importantly, *AVP1/LtRCA* co-overexpressing plants had higher plant biomass and significantly higher seed yield under combined heat and drought stress conditions than *AVP1*-overexpressing and *LtRCA*-overexpressing plants, indicating that co-overexpression of *AVP1* and *LtRCA* in Arabidopsis appears to have a synergism that makes transgenic plants performing better than overexpression of these two genes separately. Our research showed a great potential of improving plant productivity by using a thermo-stable RCA to maintain photosynthetic rate at elevated temperatures, which could translate into higher crop yield when climate change brings us a more serious problem in the future. Our proof-of-concept work in Arabidopsis has been confirmed recently as our preliminary work with transgenic cotton indicate that *AVP1/LtRCA* co-overexpressing cotton plants perform much better under combined heat and drought stress conditions in both greenhouse and field trials than wild-type and *AVP1*-overexpressing cotton plants, with significantly improved fiber yields under all stress conditions tested (unpublished data).

## Future of RCA

Several fundamental details remain to be explicated on the interaction between RCA and Rubisco including the role of ATP hydrolysis during Rubisco activation, the exact conformational changes during Rubisco activation and inactivation, and factors contributing to the inactivation and activation of Rubisco by RCA. Additionally, the regulation of RCA requires further exploration to better understand its catalytic activity and how it associates with the chloroplast thylakoid membranes. RCA abundance shifts between thylakoid membranes and chloroplast stroma during exposure to heat stress (Jin et al. 2006), implying that changes take place in the binding capacity of RCA to membranes under heat stress conditions. Several mechanisms on the regulation of RCA activity have been proposed including phosphorylation, acetylation and redox balance (Kim et al. 2019). However, deciphering the factors involved in driving these regulatory activities and their interaction in this complex network, requires further exploration. With the identification and characterization of RCA-like proteins in lower organisms such as cyanobacteria might shed some light in our understanding of the importance of activase and its evolution towards its role in Rubisco activation (Flecken et al. 2020). Further, in evaluating the effects of overexpressing *RCA*, the developmental stage of leaves as well as the ratio of Rubisco vs RCA appear to be important in achieving the optimal photosynthetic efficiency. It is well established that Rubisco inactivation is a primary reason for the reduced net photosynthesis in plants, which could be due to limitation of RCA as well as RCA-independent activities under heat stress conditions (Kim and Portis Jr 2005). In this context, a great emphasis is placed on research related to the 3′-UTR regions of *RCA* transcripts as a major determinant in conferring thermo-tolerance (Deridder and Salvucci 2007; Deridder et al. 2012). Moreover, an acidic minipeptide designed to resemble the conserved C-terminal extension of RCA was fused to several heat labile proteins such as cytosolic ascorbate peroxidase and TATA-box binding protein, and these fusion proteins were introduced into *Escherichia coli* and *Saccharomyces cerevisiae* cells to evaluate the thermo-stability of these recombinant proteins (Zhang et al. 2015). The positive results of improved thermo-stability in these fusion proteins suggest that this strategy in increasing RCA thermo-tolerance might improve crop performance at elevated temperatures.

The increased Rubisco inactivation under heat stress as a consequence of reduced RCA activity should be well examined. For instance, this could be a result of a protective mechanism against photorespiratory metabolites that prevent further damages to plant cells under heat stress (Sharkey 2005; Weston et al. 2007). If a single amino acid substitution could improve the thermo-tolerance of RCA, the question arises why this beneficial feature did not take place under natural selection in nature. At elevated temperatures, the oxygenase activity of Rubisco also increases, leading to carbon loss and increased energy demands (Mueller-Cajar and Whitney 2008). On the other hand, reducing the Rubisco activity to minimize photorespiration even under moderate heat stress could negatively affect plant growth and survival. However, the initial work by Kumar et al. (2009) and Kurek et al. (2007) clearly indicated that improving the thermo-tolerance of RCA could be achieved, while maintaining high Rubisco activity and net photosynthesis, thereby leading to improved plant growth and development under moderate heat stress conditions.

Moreover, the negative effect caused by increasing RCA content such as decreased amount of Rubisco, leading to reduced photosynthesis and biomass (Fukayama et al. 2012; Suganami et al. 2020), still remains a topic of debate that requires further evidence. Research has shown how a diverse collection of factors apart from RCA markedly affect Rubisco content in leaves, including atmospheric CO_2_ level, plant hormones such as ABA, removal of fruit altering the sink-capacity, and rain (Crafts-Brandner et al. 1991; Fukayama et al. 2010; Fukayama et al. 2018). Hence, if the photosynthetic capacity needs to be improved and the yield increased, the underlying complex mechanism regulating Rubisco and RCA contents require to be carefully assessed. Experiments would need to be carried out under heat stress conditions as well as in different crops before a clear conclusion can be drawn.

Species specificity is also a factor that should be considered when overexpressing a foreign *RCA* gene. Although numerous reports showed that RCA and Rubisco from two different plant species could interact with each other (Fukayama et al. 2012; Yamori et al. 2012; Wijewardene et al. 2020), incompatibility could occur with certain species, which could pose a serious problem. For instance, an earlier work showed the specificity of RCA-Rubisco interaction between Solanaceae plants like tobacco and non-Solanaceae plants such as spinach, making RCA incapable of activating Rubisco (Wang et al. 1992). This is primarily due to the differences in amino acid residues in the interaction domains of RCA and Rubisco, which are essential for the correct protein-protein interaction (Ott et al. 2000; Kumar et al. 2009), resulting in less effective enzyme-substrate association (Li et al. 2005; Hasse et al. 2015). The coevolving mechanism of RCA and its Rubisco leads to a highly species-specific recognition between RCA and Rubisco (Bhat et al. 2017a; Nagarajan and Gill 2018). To overcome such obstacles, in addition to the studies related to protein structure and biochemical characteristics, further research should be carried out to explore the existence of thermo-tolerant varieties of the same plant species where thermo-tolerant RCA varieties could be introduced to thermo-susceptible cultivars. Research related to wheat (Scafaro et al. 2019) and rice (Scafaro et al. 2016; Scafaro et al. 2018) have shown the presence of several wild species thriving in higher environmental temperatures, whose RCA could be introduced into the commercial varieties to enhance RCA’s thermo-stability in wheat and rice. At certain times, a lower catalytic activity of RCA is observed among the thermo-tolerant species despite their thermal stability, however, this drawback appears to be compensated by the higher RCA/Rubisco ratio in leaves (Scafaro et al. 2016). Careful assessment of thermostable as well as sensitive RCA and their regulatory sequences such as promoter regions may provide useful insights into this avenue to better understand, manipulate, and create RCA catering to the prevailing agricultural requirements.

Countless studies demonstrated the important role played by RCA and how it could be utilized to effectively improve the thermo-tolerance of plants, especially agriculturally important crops. In addition to heat tolerance, several studies reported the importance of RCA in conferring tolerance to low temperatures and low light (Jurczyk et al. 2015b; Bi et al. 2017), suggesting that there is much to be learned about this molecular chaperone. Apart from genetic manipulation, numerous methods of increasing RCA content in plants have been tried, including application of chemicals and creating mini peptides. For example, upon exogenous application of sodium bicarbonate on rice leaves, it was observed that RCA transcript and protein levels were significantly up-regulated and biochemical parameters such as RuBP regeneration, net photosynthetic rate, and initial Rubisco activity were also enhanced (Chen et al. 2014). Likewise, pretreatment of wheat seedlings with 2,4-epibrassinolide (a type of the steroid hormone brassinosteroids) increased the initial Rubisco activation and RCA content under heat stress and heat plus drought stress conditions (Zhao et al. 2017). Moreover, studies on RCA have expanded beyond the target of just improving crop productivity. For instance, research was carried out on overexpressing a gene encoding a RCA-like protein in the microalgae *Nannochloropsis oceanica* in the prospects of improving biomass, which could lead to better carbon sequestration and higher biofuel production (Wei et al. 2017). Thus, it is evident that research pertaining to RCA not only benefits the global agricultural production, but also broadens to cover a wide range of areas that would help many other industries increasing productivity through an environmentally friendly approach.

In addition to the biochemical and genetic engineering works, the availability of whole genomes of many plant species, multiple databases, bioinformatics tools that allow the prediction of gene and protein structures and functions, and the Next Generation Sequencing through transcriptome data analysis, has aided in accelerating the process of identifying and predicting novel and putative heat responsive genes in plant germplasms (Kumar et al. 2017; Nagarajan and Gill 2018). This knowledge could also help us understand the structural and evolutionary changes occurred in different RCA isoforms that have similar or different functions, while enables certain RCAs like tobacco RCA that is a single isoform, to carry out activities of both long and short isoforms. The expression QTL (eQTL) mapping was carried out for maize RCA where the transcript and protein abundance under different growing seasons were evaluated and stably expressed eQTLs were identified that are related to maize RCA expression (Sun et al. 2017). The eQTL is a great tool in identifying factors that influence gene expression *in vivo*, where these determinants could be classified as *cis*- or *trans*-acting factors based on the proximity of eQTL to the gene of interest (Druka et al. 2010). Thus, the accessibility to information through these emerging methods enables us to better understand the structure and regulatory components of RCA in designing a more thermo-tolerant RCA with better Rubisco activation properties, as well as identifying important molecular markers for thermo-tolerant traits in plant breeding (Sun et al. 2017; Kumar et al. 2019), assisting to mitigate the thermo-lability of RCA and produce climate-smart crops. Different molecular targets could be used in increasing plant photosynthesis under heat stress, since parameters such as species, growth conditions, and climate participate in determining the limiting step of photosynthesis under stressful conditions (Yamori et al. 2014). Ogbaga et al. (2018) discussed the potential use of RCA from thermophilic cyanobacteria in the hope of expanding the thermo-tolerance window in agricultural crops without the expense of additional energy input for photorespiration, suggesting that this could be another area worth of further exploring.

**Fig. 3:**
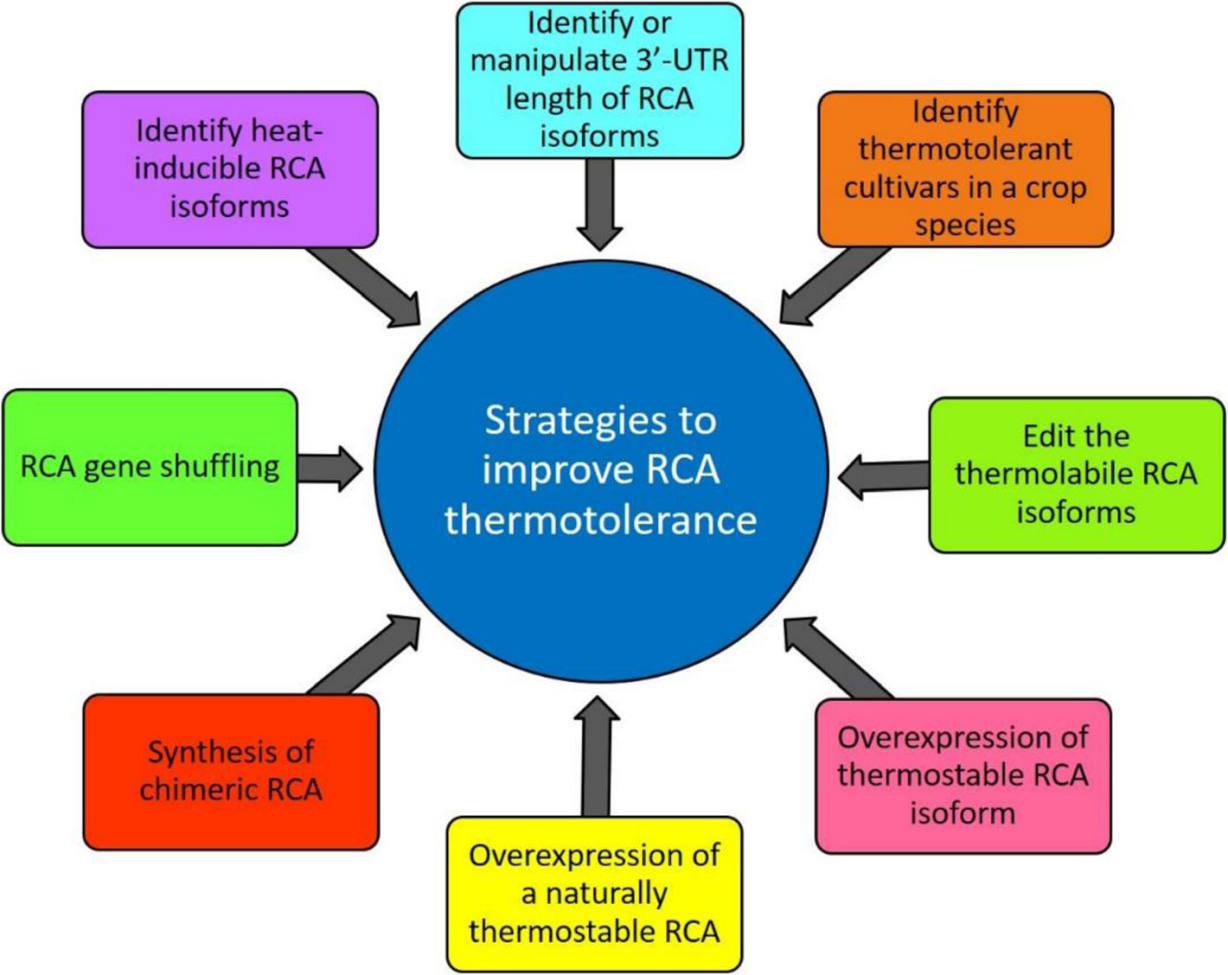
Potential strategies of using RCA to improve photosynthesis at elevated temperatures, which could increase crop yield under the environmental conditions un-avoidably brought by the climate changes in the future

In summary, RCA is one of the critical factors in photosynthesis and is a key that determines crop yield under heat stress conditions. As climate change evolves, RCA will emerge as a great tool for keeping food production in line with the world population growth in the future. While many methods are being tested in keeping RCA fully functional at elevated temperatures (Fig. 3), some appear to be very successful including the use of *LtRCA* that encodes a naturally thermo-tolerant RCA (Wijewardene et al. 2020). Although these efforts in understanding how RCA maintains its activity at elevated temperatures and how RCA interacts with Rubisco at the molecular level must be continued, the thermal properties of RCA and its ability to respond to changing temperatures require substantial attention, and the genetic engineering of RCA in crops must ensure that enhancing RCA temperature resilience would not adversely affect its interaction with Rubisco, which will ultimately pave the way to improve plant biomass and yield under prevailing environmental conditions, thus safeguarding global food security in the future.

## Data Availability

Not applicable.
